# Distinct Metabolites in Osteopenia and Osteoporosis: A Systematic Review and Meta-Analysis

**DOI:** 10.3390/nu15234895

**Published:** 2023-11-23

**Authors:** Yuhe Wang, Xu Han, Jingru Shi, Zeqi Liao, Yuanyue Zhang, Yuanyuan Li, Miao Jiang, Meijie Liu

**Affiliations:** 1 Beijing Key Laboratory of Traditional Chinese Medicine Basic Research on Prevention and Treatment for Major Diseases, Experimental Research Center, China Academy of Chinese Medical Sciences, Beijing 100700, China; 18801094991@163.com (Y.W.); shijingru2023@163.com (J.S.); 13611244269@163.com (Z.L.); zhangyuanyue0306@163.com (Y.Z.); 18291830737@163.com (Y.L.); 2 Institute of Basic Research in Clinical Medicine, China Academy of Chinese Medical Sciences, Beijing 100700, China; zyrhanxu@163.com

**Keywords:** osteoporosis, osteopenia, distinct metabolites, metabolomics

## Abstract

Multiple studies have indicated that distinct metabolites are involved in the occurrence and development of osteopenia (ON) and osteoporosis (OP); however, these metabolites in OP and ON have not yet been classified and standardized. This systematic review and meta-analysis included 21 articles aiming to investigate the distinct metabolites in patients with ON and OP. The quality of the included articles was generally high; seventeen studies had >7 stars, and the remaining four received 6 stars. This systematic review showed that three metabolites (phosphatidylcholine (PC) (lipid metabolites), galactose (carbohydrate metabolites), and succinic acid (other metabolites)) increased, four (glycylglycine (gly-gly), cystine (amino acids), sphingomyelin (SM) (lipid metabolites) and glucose (carbohydrate metabolites)) decreased, and five (glutamine, hydroxyproline, taurine (amino acids), lysophosphatidylcholine (LPC) (lipid metabolites), and lactate (other metabolites)) had conflicting directions in OP/ON. The results of the meta-analysis show that gly-gly (MD = −0.77, 95%CI −1.43 to −0.11, *p* = 0.02) and cystine (MD = −5.52, 95%CI −7.35 to −3.68, *p* < 0.00001) decreased in the OP group compared with the healthy control group. Moreover, LPC (MD = 1.48, 95%CI 0.11 to 2.86, *p* = 0.03) increased in the OP group compared with the healthy control group. These results indicate that distinct metabolites were associated with ON and OP, which could be considered a predictor for OP.

## 1. Introduction

Osteoporosis (OP) is a systemic metabolic bone disease characterized by low bone mineral density (BMD) and the destruction of bone microstructure, which can increase the risk of fractures [1]. Osteopenia (ON) is a stage between the normal BMD and OP, where bone density is suboptimal but still not as low as OP [2]. Without adequate treatment, ON tends to develop into OP. There are some 200 million OP patients in the world, which is expected to reach 400 million in the next decade [3]. In addition, 20% of people aged > 50 experience OP-related fractures, among which hip fracture is the most devastating [4]. By 2020, the number of ON cases has reached 47 million [5]. It has been estimated that the medical expenses for OP fractures in China will reach CNY 132 billion by 2035, while this portion of healthcare expenditure may climb to CNY 163 billion yuan by 2050 [6].

Currently, OP and ON are mainly diagnosed according to BMD value by dual-energy X-ray absorptiometry (DXA) [7,8], and these diseases are considered preventable and treatable. The mainstream therapies include estrogen treatment, alendronate sodium, calcium supplementation, and Chinese medicine [9]. Proper treatment is believed to effectively decrease the risk of fracture even for those who have already suffered from brittle fracture [10], and the key point for effective treatment is early diagnosis. For example, as one of the mainstream therapies, calcium supplementation can hardly restore BMD to normal levels and has weak effects on fractures [11]; however, it is effective as an early preventive measure [12]. Should the diagnosis be made as early as possible, the treatment effectiveness and cost would both be satisfying. Therefore, an effective predictor, or a biomarker, which can warn the potential of the diseases, is warranted.

Generally, biomarkers are often discovered based on the mechanisms of disease [13]. OP is considered to be the disorder of bone homeostasis, which means the imbalance between the bone formation mediated by osteoblast (OB) and the bone resorption mediated by osteoclast (OC) [14]. Nonetheless, the specific mechanism of bone homeostasis disorder remains unclear. Multiple factors have been proven to contribute to OP, such as age, gender, hormone level, use of oral glucocorticoids, etc. [15]. Older individuals, especially postmenopausal women, are the most affected by ON or OP [16]. Respectable efforts have been made to construct predictive models using these factors; however, there are no large-scale clinical applications for these models [17].

Great expectations have been placed on bone turnover markers (BTMs), including procollagen type I amino-terminal peptide (PINP) and C-terminal telopeptide of type I collagen (CTX-I), in the monitoring and treatment of OP and ON [18]. Nevertheless, BTMs are proteins or matrix-degradation products produced and released by OB and OC, so they cannot exert an early predictive role before the functional abnormalities of OB and OC occur [19]. Due to this natural variability, their independent prognostic value for OP is uncertain [20]. Therefore, the proper candidates for the predictive biomarkers of OP and ON are still an issue worth considering.

In recent years, the role of metabolites in OP or ON has been considered [21], as the disordered body metabolism can affect bone metabolism, leading to the decrease in BMD and causing OP [22]. Since OP and ON are metabolic diseases, and given the rapid progress in metabolomic technique, it is possible and reasonable to elucidate the characteristics of the disease status by using some or a pattern of metabolites in the body.

Metabolomics, a popular method for systematically profiling small molecules, is widely used in various metabolic diseases [23]. This approach can dynamically reflect the small molecule metabolites and their changes in organisms [24]. Increasing evidence has indicated that the altered metabolites relate to the ON/OP [25], which provided novel insights into a prediction of OP and highlighted the potential of metabolites as markers for OP. Recently, preclinical and clinical studies have proved that distinct metabolites can be used to help predict ON/OP and for monitoring during the treatment of OP [26,27,28]. Moreover, it may provide a feasible solution to perform the differential metabolite between the OP or ON and healthy participants. However, the distinct metabolites in OP and ON have not yet been classified and standardized.

The present systematic review and meta-analysis aimed to summarize the differential metabolites in OP and ON patients compared with healthy people, to establish the metabolic pathways associated with OP/ON, and further discuss their potential in predicting OP so as to facilitate a new perspective for the early identification and prevention of OP in clinical practice.

## 2. Materials and Methods

Our systematic review and meta-analysis adhered to the standard criteria PRISMA [29]. This research protocol has been registered in the PROSPERO registry (ID = CRD42022365040).

### 2.1. Search Strategy

PubMed, Web of Science, Embase, the Cochrane Library, the Chinese databases of China National Knowledge Internet (CNKI), and the Wan-fang database were searched for relevant articles published from their inception up to 5 August 2022 without restrictions on countries or article type. Our search terms combined interventions (Metabolomics) with diseases (ON, OP) in humans. The detailed search strategy is shown in Table S1.

### 2.2. Eligibility Criteria

Inclusion criteria were the following: (1) Human study; (2) OP and ON were defined by measuring BMD; (3) Randomized controlled trial, case-control study, cross-sectional study; (4) Metabolites were identified by metabolomics technique in blood or urine samples; (5) published in English or Chinese language. Reviews, studies on children, adolescents, and pregnant women, secondary OP, subjects with other basic diseases aside from OP, patients receiving treatment that affected the metabolite level within three months, and non-original articles were excluded.

### 2.3. Study Selection

Firstly, we removed duplicates, after which the titles and abstracts of the remaining articles were screened separately by two researchers, and the studies that did not meet the predefined criteria were excluded. Moreover, the full text of the eligible articles was subjected to further screening. Any disagreements were discussed with the third researcher to minimize selection bias.

### 2.4. Data Extraction

Two independent researchers extracted information from eligible articles, including the first name of the author, year of publication, study design, number of OP or ON and control, age, body mass index (BMI), metabolomics technique, biological sample, the name of different metabolites, the variation trend, and the concentration of metabolites and associated metabolic pathways. The data were shown in graphs, and WebPlotDigitizer-4.5 was used to extract data from graphs. Disagreements were resolved through discussion and consensus was reached with a third investigator. Classify different metabolites according to amino acid, lipid, carbohydrate, and other metabolites.

### 2.5. Quality Assessment

We evaluated and scored the quality of the case-control studies by the Newcastle–Ottawa Scale (NOS), including whether the sample selection was random, comparability of cases and controls, and exposure [30]. Among these categories, each item in selection and exposure could obtain at most one point, while in comparability, it could obtain up to two points. The quality assessment of the cross-sectional study was conducted by using a modified Newcastle–Ottawa Quality Assessment Scale [31], which includes the following items: (1) Selection (composed of three items): representativeness of the sample, sample size, and non-response rate; (2) Comparability (one item): between respondents and non-respondents; (3) Outcome and Analysis (four items): assessment of outcome of hypersensitivity, reporting of point estimate (prevalence), reporting of the measure of variability for the point estimate and accounting for correlation between multilevel units. Two independent researchers implemented the quality assessment, and arbitration was conducted by the third researcher in case of disagreement.

### 2.6. Statistical Analysis

A qualitative analysis was performed for the different metabolites by counting the frequency across the included studies. For the concentrations of different metabolites, a meta-analysis was conducted across studies using the MD with the confidence intervals 95% (95%CI) when 2 different studies reported the same metabolites. Finally, a random effect model was applied when the heterogeneity was high (I2 > 50%); conversely, the fixed effect model was applied. Sensitivity analyses were performed to investigate the influence of possible bias by removing studies one by one.

## 3. Results

### 3.1. Literature Search Results

**Figure 1:**
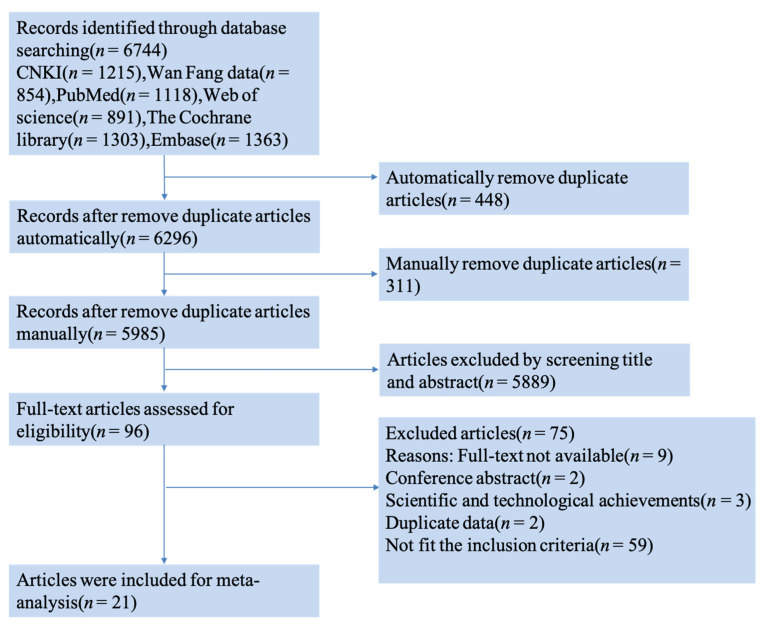
Flowchart of literature screening.

The literature search and selection of articles are shown in Figure 1. A total of 6744 articles were found in Chinese and English databases, 759 of which were deleted due to the repetitive content. Among the 5985 titles and abstracts that were reviewed independently by two researchers, 5889 were excluded. After reading the complete text, 75 articles were excluded for various reasons (Figure 1). Finally, 21 articles, 4 in Chinese and 17 in English, were included.

### 3.2. Characteristics of Included Studies

All the studies were published between 1997 and 2022, including 20 case-control studies and one cross-sectional study. Among these, one study [32] focused on ON as the target disease, six studies [27,33,34,35,36,37] focused on both ON and OP, while the remaining fourteen studies [38,39,40,41,42,43,44,45,46,47,48,49,50,51] concentrated on OP. Notably, there were five studies [38,48,49,50,51] on OP that primarily focused on the differentiation of traditional Chinese medical (TCM) syndromes in their study design. A total of 3721 participants were recruited from China, Hungary, Japan, Jordan, Brazil, Korea, and the USA. In addition, 15 studies reported on age and BMI, and six studies [39,41,42,44,47,51] failed to record BMI. The included studies analyzed the metabolites from blood and urine samples via GC-MS, LC-MS, and HNMR. The characteristic of each study is shown in Table 1.

### 3.3. Risk of Bias of Included Studies

The risk of bias in case-control studies was assessed by NOS, and the cross-sectional study was conducted using a modified Newcastle–Ottawa Quality Assessment Scale. The quality of seventeen studies was >7 stars, and that of the four other studies equaled 6 stars. The maximum score for NOS is 9 stars and the minimum score for NOS is 6 stars. The risk of bias evaluation of the included studies is shown in Table 1. The assessment details for every item are listed in Table S2.

### 3.4. Qualitative Synthesis

In this systematic review and meta-analysis, we collected many different metabolites, including amino acid metabolites, lipid metabolites, carbohydrate metabolites, and other metabolites in ON/OP. Distinct metabolites identified in ON and OP are shown in Table 2. We have provided a list of abbreviations in Table S3.

### 3.5. Amino Acids

More than 10 studies compared the differences in amino acid levels between ON/OP and healthy subjects. In particular, a study carried out by Zhao et al. [45] evaluated the association between BMD and amino acids, reporting that seven amino acids and their derivatives were significantly associated with low BMD. Moreover, a study conducted in China that included 701 participants [27] found that 13 amino acids were the risk factors for ON/OP through the LC-MS metabolomics method. At least five studies detected the level of glutamine (which can be converted to glutamic acid in the body) and glutamic acid and analyzed the relationship between glutamine or glutamic acid and BMD. Three studies [45,46,47] reported a negative correlation between glutamine and BMD; however, these results contradict those of another study [33] in which the glutamine level declined in the participants with ON or OP compared with the healthy controls. Interestingly, Wang et al. [33] also found that the concentration of glutamine was elevated in the OP group compared to the ON group. Among four studies [33,40,41,47] that analyzed the relationship between hydroxyproline and BMD, three found significant negative associations, unlike the study of Deng et al. [41]. Miyamoto and Kou [40,43,47] observed that gly-gly and glycine levels were significantly lower in the low BMD group than in the high BMD group. Furthermore, the concentrations of cystine and cysteine were decreased in the individuals with OP [40,45,47], which suggested that cysteine may also be a protective factor for BMD. Two studies [35,37] revealed that the taurine level declined in patients with low BMD, contrary to another study [45].

### 3.6. Lipid Metabolites

The correlations between lipid metabolites and ON/OP were also analyzed.

Eleven studies focused on lipid metabolites, seven of which evaluated the levels of phosphatidylcholine (PC) and its metabolite lysophosphatidylcholine (LPC) in different participants. Three case-control studies [34,42,43] revealed a negative correlation between serum PC and BMD; two other studies [41,42] discovered a significantly lower level of LPC in the OP group than in the normal BMD group. However, three studies [33,43,47] reported opposite results, where the LPC level was higher in the low BMD group than in the normal group.

More than 10 differentially expressed phospholipids, including sphingomyelin (SM), cardiolipin (CL), phosphatidic acid (PA), glycerophosphate, etc., identified by high-throughput techniques, were found in the ON/OP group. Among four studies that determined the level of SM, two [42,43] suggested a positive correlation between SM and BMD.

In addition, two studies [34,43] reported a close correlation between low carbon number-saturated lipids (palmitic acid, stearic acid) and OP.

### 3.7. Carbohydrate Metabolites

Sugar metabolites, including galactose, glucose, and fructose, were closely correlated with the prevalence of ON/OP. Two Chinese studies [43,46] reported lower glucose levels and one study [37] indicated a higher galactose concentration in the OP group than in the control group.

### 3.8. Other Metabolites

Organic acids: at least nine analyses recruited organic acids. The determination in 74 individuals with OP and 78 control subjects [35,43] showed that the lactate level was significantly higher in OP than in the control group. However, an opposite result was also reported [46]. Succinic acid was indicated as an increased risk for low BMD [37,41,45].

Acylcarnitines: C5-DC, C3-DC-M, acetylcarnitine, and 3-hydroxy-11-octadecenoylcarnitine were associated with ON/OP [33,34,45]; however, there was little overlap among these studies.

Urea cycle metabolites, ornithine, citrulline, and arginine, were considered OP risk factors [27,33,43,47].

Bile acids: two studies [41,45] identified more than eight bile acids (BAs) and the detected BAs were associated with OP.

Other compounds, steroids [32,39], purines [27], neonicotinoids, and their characteristic metabolites [44] were associated with ON/OP in isolated reports.

### 3.9. Metabolites and Traditional Chinese Medicine Syndrome

**Figure 2:**
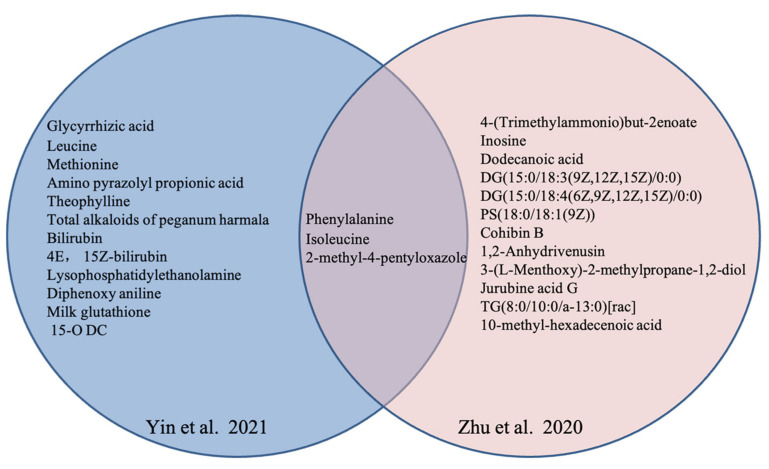
Distinct metabolites in OP with Yin deficiency of liver and kidney in different studies [48,49].

**Figure 3:**
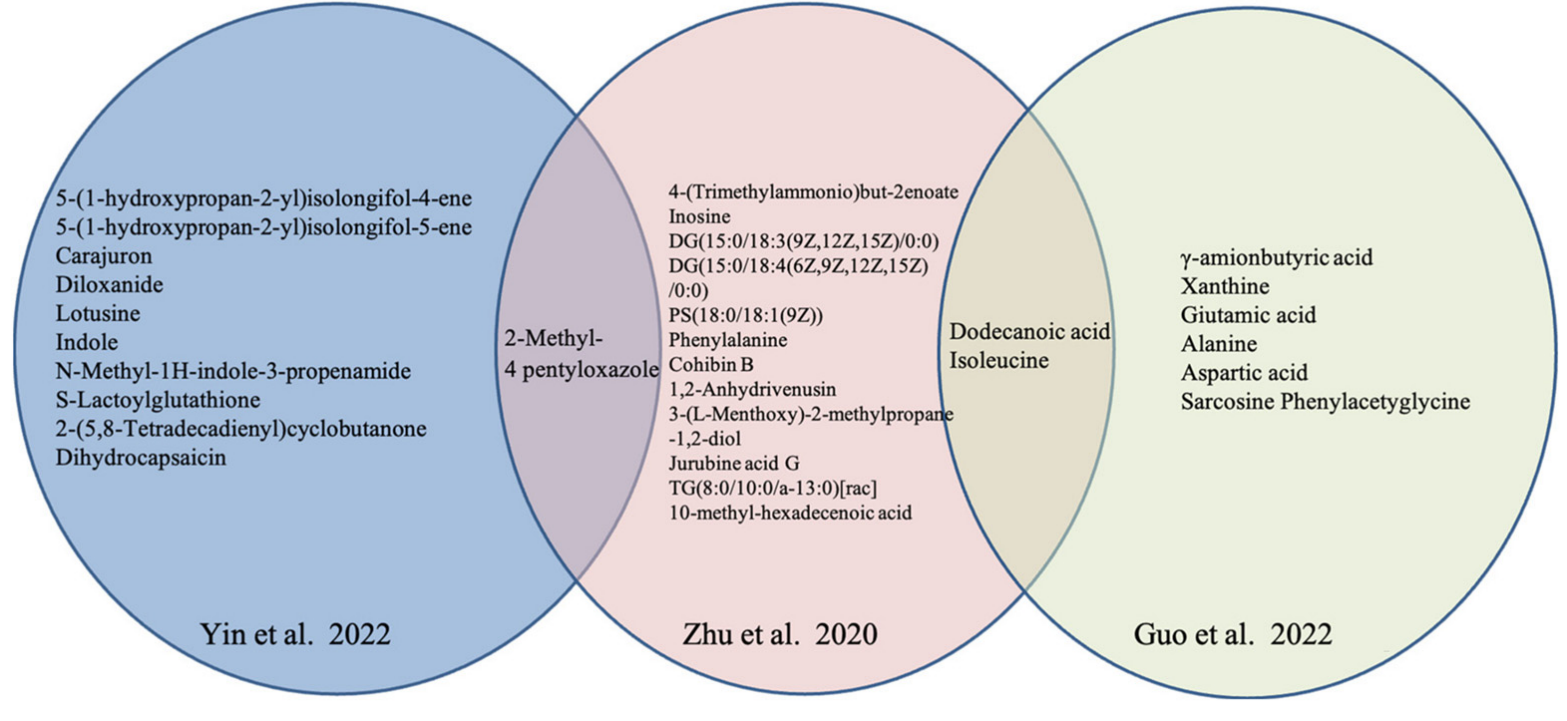
Distinct metabolites in OP with Kidney-Yang deficiency in different studies [38,49,51].

Notably, four studies [48,49,50,51] in Chinese and one in English language [38] explored the potential distinct metabolites in OP with TCM syndrome. Multiple differential metabolites, including amino acids (phenylalanine, isoleucine), oxazole (2-methyl-4-pentyloxazole), and organic acids (dodecanoic acid) were anchored to characterize the various TCM syndrome of OP from healthy subjects. The distinct metabolites in Yin deficiency of liver and kidney syndrome are shown in Figure 2, and those in Kidney-Yang deficiency syndrome are listed in Figure 3.

### 3.10. Pathways Analysis

Some of the included studies described metabolic pathways with ON/OP. As depicted in Table 3, 10 studies [34,37,41,42,43,45,47,48,50,51] analyzed differential pathways related to ON/OP, including amino acids metabolism pathways (e.g., valine, leucine, and isoleucine biosynthesis and degradation, tryptophan metabolism, histidine metabolism, taurine metabolism), and lipid metabolism pathways (e.g., glycerophospholipid metabolism, biosynthesis of unsaturated fatty acids). In addition, Li et al. [50], Aleidi et al. [34], and Zhao et al. [45] certified that the pathways of the aminoacyl-tRNA biosynthesis were differently expressed in the group of ON/OP. The TCA cycle was the most important metabolic pathway related to ON/OP found by Yu et al. [37] and Miyamoto et al. [47]. Finally, several studies found that choline metabolism, acetaldehyde, dicarboxylate, and glucose metabolism were expressed differently in the ON/OP.

### 3.11. Meta-Analysis for Metabolites

**Figure 4:**
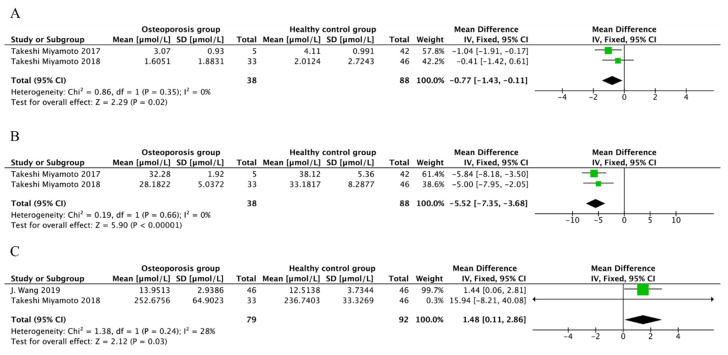
Forest plot for different metabolites (μmol/L). (**A**) Forest plot of the concentration of gly-gly in OP patients and controls. Data from references [40,47]. The level of gly-gly decreased in OP group compared to the control group. (**B**) Forest plot of the concentration of cystine in OP patients and controls. Data from references [40,47]. The results reveal that the level of cystine decreased in OP group compared to the control group. (**C**) Forest plot of the concentration of LPC in OP patients and control. Data from references [33,47]. The level of LPC was higher in OP group than the control group. Note: The green boxes represent the point estimates for each study, and the black boxes represent the combined values of the study results.

A metabolite was included in the meta-analysis if at least two studies reported its concentration with the variables of mean and SD. Due to the limited consistency of the included studies, three metabolites, including gly-gly, cystine, and LPC, were included in the meta-analysis. The results reveal that the level of gly-gly (MD = −0.77, 95%CI −1.43 to −0.11, *p* = 0.02) and cystine (MD = −5.52, 95%CI −7.35 to −3.68, *p* < 0.00001) decreased in the OP group compared to the control group, without heterogeneity among the included studies (I2 = 0%, *p* > 0.1) (Figure 4A,B). The level of LPC was higher in the OP group than in the control group (MD = 1.48, 95%CI 0.11 to 2.86, *p* = 0.03) (Figure 4C). Due to the low degree of heterogeneity, a fixed effect model was used for meta-analysis.

## 4. Discussion

This systematic review and meta-analysis identified a number of blood or urine metabolites related to ON/OP in humans. In addition, this systematic review detected several metabolic pathways associated with OP/ON, such as amino acids metabolism pathways, lipids metabolism pathways, and the TCA cycle. Moreover, this systematic review also analyzed potential distinct metabolites in OP with different TCM syndromes.

Studies found that amino acid metabolism can regulate bone metabolism and is involved in the BMD [52,53,54]. Amino acids may directly regulate the proliferation, differentiation, and apoptosis of OB and OC [55,56]. Certainly, amino acids also affect OB and OC through their actions on the secretion of insulin-like growth factor 1 (IGF-1) [57]. In addition, amino acids influence BMD by affecting the intestinal absorption of calcium [58]. As one of the amino acids, proline has a positive association with BMD, a derivative that can increase serum estradiol and alkaline phosphatase [59]. Some studies have reported on the role of hydroxyproline in the stability of collagen [60]. However, the present meta-analysis did not determine a definitive association between hydroxyproline and BMD. Notably, in the study of Wang et al. [33], the level of hydroxyproline in ON was elevated while it declined in OP compared to the normal controls, which may indicate that hydroxyproline is associated with the severity of ON. Therefore, more studies are needed to clarify this association. Moreover, tyrosine, which is present in thyroid hormones, has been proven to exert a protective effect against OP and can stimulate the expression of OB for the production of collagen [61]. Unfortunately, in the present work, only one study reported the correlation between BMD and the level of tyrosine. Furthermore, cystine and cysteine could accelerate bone regeneration by activating OB differentiation [62]. In this work, we demonstrated a positive association between cystine and BMD. Some previous studies reported on the potential role of glutamine and glutamic acid in bone metabolism. Glutamate receptors can be expressed in bone cells, and their signaling can regulate bone remodeling [63]. In addition, glutamine can provide the needed energy for bone resorption of OC through the TCA [64]. However, this meta-analysis has not found a definitive association between glutamic acid and BMD due to the lack of reports on the concentration values of included studies. In our meta-analysis, we detected a significant difference in gly-gly levels between individuals with OP and healthy individuals. Compared with the healthy control group, the gly-gly level was decreased in the OP group, which indicated that gly-gly could predict low BMD in OP.

Lipidomics has suggested that a number of lipids may be predictive of ON/OP. Moreover, many studies have found that lipid metabolism disorders may have a certain relationship with the incidence of OP [65,66]. Phospholipids are the main component of bio- membrane. The articles included in this systematic review reported that more than 10 phospholipids (e.g., SM, CL, PA, glycerophosphate), identified by high-throughput techniques, were distinct in those with and without ON/OP. SM can generate ceramides under hydrolyzed, which produces reactive oxygen species, thereby inhibiting bone formation and leading to the occurrence of OP [67]. In this study, at least two articles suggested a positive association between SM and BMD, where the level of SM was decreased under hydrolyzed, thus leading to OP, closely related to inflammatory reactions [68]. Triglycerides (TGs) and diacylglycerols (DGs) have been reported to regulate inflammation reactions, promoting the expression of inflammatory factors such as IL-6, IL-1β, and IL-8 and inducing oxidative stress and OP [69]. However, included studies reported inconsistent results, thus suggesting that further research is still needed. As a metabolite of PC, LPC plays a crucial role in the occurrence of OP. On the one hand, LPC is the component of oxidized low-density lipoprotein (ox-LDL), which can facilitate the differentiation from bone marrow mesenchymal stem cells (BMSCs) to adipocytes and inhibit BMSCs in the differentiation from BMSCs to OB, thus accelerating the occurrence of OP [42]. On the other hand, LPC has also been identified as a potential OC differentiation factor [70]. More importantly, LPC can increase the concentration of free calcium in intracellular [71] and reduce the concentration of calcium in bone, leading to OP. Our meta-analysis also demonstrated a significantly negative association between the concentration of LPC and BMD, which is consistent with the previous findings. BAs were reported to be associated with ON and OP. A recent study suggested that sodium deoxycholate reduces intestinal calcium absorption, whereas lithocholic acid stimulates calcium absorption [45]. UCDA, a secondary bile acid, can promote OB differentiation and bone mineralization. However, different studies reported inconsistent results, suggesting that the type of BAs should be considered when the relationship between BAs and BMD has already been analyzed.

In our study, the sugars (e.g., galactose, glucose, and fructose) were associated with BMD, which may be partly explained by abnormal energy metabolism affecting OP [72]. Moreover, our research found that organic acids like succinic acid had an increased risk for low BMD. Succinic acid is important in mitochondrial function, and mitochondrial dysfunction is related to age-related diseases such as OP. Moreover, succinic acid receptor activation can enhance the function of OC, leading to a decrease in bone mass.

This research found that amino acid metabolism had an extreme difference in OP with Yin liver and kidney deficiency. The metabolic state of different syndrome types of TCM requires different energy, which leads to changes in amino acid metabolism. Organic acids and their metabolites showed apparent differences in the OP group with Kidney-Yang deficiency compared with the healthy group. According to up-to-date research, Kidney-Yang deficiency is closely related to the neuroendocrine system [73], often showing the clinical manifestations of sympathetic insufficiency, such as a pale complexion and lukewarm limbs. This may be related to the metabolic disorder of organic and amino acids, affecting the nervous system.

The strengths of the present study include that we perform an extensive literature search. We searched the main databases in English and Chinese, thus minimizing the possibility of missing any major published report. Next, to the best of our knowledge, this is the first study that performed qualitative synthesis to evaluate the relationship between distinct metabolites and the occurrence of OP. This is also the first study that investigated the relationship between distinct metabolites and OP with different TCM syndrome types, thus providing a theoretical basis for the biological nature of TCM syndrome types. Moreover, we described associated metabolic pathways in this review. Finally, the included studies were of high quality, although most results show significant heterogeneity.

Despite the novelty of our research, several limitations should be pointed out. First, most included studies reported inconsistent results, so only a few meta-analyses could be conducted. Second, menstruation and BMI are important influencing factors of OP, but we did not perform subgroup analysis to exclude the influence of both due to the limited number of articles in the meta-analysis. Third, not all trials clearly distinguished the two groups of ON and OP according to BMD, and the severity of the disease needed to be standardized. Finally, due to the limitation of the research type, our results could only provide clues for finding predicted metabolites.

## 5. Conclusions

Distinct metabolites are associated with ON and OP. Our results suggest that gly-gly and cystine negatively affect BMD. However, LPC is positively associated with the occurrence of OP, which provides evidence that metabolomics can be used as predictive biomarkers for ON and OP. In addition, this systematic review found that amino acids metabolism pathways, lipids metabolism pathways, and TCA cycle are associated with OP/ON. Moreover, this systematic review also demonstrated that phenylalanine, isoleucine, and 2-methyl-4-pentyloxazole were closely linked to OP with Yin deficiency of the liver and kidney, while dodecanoic acid, isoleucine, and 2-methyl-4-pentyloxazole had a relationship with OP with Kidney-Yang deficiency. However, we found only possible candidates for diagnostic purposes in this work. The associations between metabolites and BMD should be further investigated and verified by future high-quality and large prospective cohort studies.

## Figures and Tables

**Table 1:** The characteristics and NOS of the included studies.

References	Country	Study Design	No. of OP or ON/Control	Age of OP or ON/Control (years)	BMI of OP or ON/Control (m/kg^2^)	Technique	Biological Sample	Key Findings		NOS
Yin et al., 2021 [48]	China	case-control	30 OP vs. 30 control	66.1 ± 7.5/64.3 ± 6.3	24.34 ± 2.99/24.11 ± 2.87	UPLC/MS	blood	15 different metabolites in OP with Yin deficiency syndrome: Glycocholic Acid, Bilirubin, Diloxanide, etc.		7
Zhu 2020 [49]	China	case-control	30 OP(A) vs. 30 OP(I) 30 control	65.47 ± 7.54/66.1 ± 7.47 /55.97 ± 9.47	22.93 ± 3.42/24.34 ± 2.99 /24.11 ± 2.87	UPLC/MS	serum	15 different metabolites	10 (↑) *: Inosine, Lucidenic acid G, etc.	7
									5 (↓) *: Dodecanoic acid, Cohibin B, etc.	
Li 2020 [50]	China	case-control	120 OP vs. 18 control	46–87	14.69–33.33	HNMR	serum	20 different metabolites: Glutamine, Leucine, etc.		6
Guo et al., 2022 [51]	China	case-control	20 OP vs. 12 control	62.7 ± 2.2/47.5 ± 5.4	NA/NA *	UPLC/MS/MS	serum	157 different metabolites	93 (↑): L-isoleucine, γ-Aminobutyric acid, etc. 64 (↓): Alanine, Glutamate, etc.	7
Yin et al., 2022 [38]	China	case-control	30 OP vs. 30 control	65.47 ± 7.54/55.97 ± 9.47	22.93 ± 3.42/24.11 ± 2.87	UPLC/MS	serum	11 potential metabolite biomarkers of KYADS: Indole, Lotusine, etc.		6
Poor et al., 2003 [39]	Hungary	case-control	11 OP vs. 13 control	53.8 ± 4.9/56.6 ± 5.7	NA/NA	capillary gas chromatography	urine	8 Urinary steroid different metabolites: Tetrahydro-corticosterone, 11-O-androsterone, etc.		6
Wang et al., 2019 [33]	China	case-control	Male: 40 OP vs. 46 ON vs. 46 control Female: 60 OP vs. 61 ON vs. 61 control	Male: 66.9 ± 2.9/67.2 ± 1.3/67.4 ± 1.4 Female: 60.7 ± 3.9/60.8 ± 4.0/60.1 ± 4.2	Male: 23.3 ± 2.5/23.4 ± 2.5/23.4 ± 2.4 Female: 26.8 ± 3.5/26.7 ± 3.5/26.7 ± 3.5	LC-MS/MS	blood	Male: 8 metabolites in males showed significant differences between the three groups Female: 12 metabolites showed significant differences between the three groups		8
Miyamoto et al., 2017 [40]	Japan	case-control	5 OP vs. 42 control	55.83 ± 3.6/56.34 ± 3.5	23.09 ± 1.8/22.25 ± 2.53	LC/MS	serum	protein metabolism	(↓) Gly-Gly, cystine (↑) hydroxyproline	6
Aleidi et al., 2021 [34]	Jordan	case-control	25 OP vs. 22 ON vs. 22 control	66.16 ± 1.78/64.64 ± 1.72 /54.82 ± 1.03	30.70 ± 1.4/30.38 ± 1.84 /32.21 ± 1.1	UPLC/MS	serum	94 dysregulated metabolites:	52 (↑) 42 (↓)	8
Deng et al., 2021 [41]	China	case-control	32 OP vs. 32 control	60.47 ± 12.39/60.59 ± 14.14	NA/NA	UHPLC-HRMS	serum	The differential metabolites	(↑) PE, TG(18:0/18:0/18:0), cyclic Melatonin, etc. (↓): LPC, 4-Hydroxyproline, etc.	9
Cao et al., 2021 [42]	China	case-control	36 OP vs. 55 control	57.51 ± 4.59	NA/NA	LC-MS	blood	10 different lipid metabolites:	6 (↑): PC (18:0/20:4), TG (16:0/10:0/20:4), CL (19:0/18:2/20:0/22:6), CL (75:4), PC (36:5), Tand G (54:4) 4 (↓): PC (36:2), CL (22:3/18:0/18:0/20:4), LPC (18:1), SM (d16:0/18:1)	7
Kou et al., 2022 [43]	China	case-control	50 OP vs. 50 control	69.3 ± 9.3/66.3 ± 10	23.8 ± 3.2/23.5 ± 4.4	GC/LC-MS	serum	18 different metabolites		8
Pontes et al., 2019 [35]	Brazil	case-control	24 OP vs. 26 ON vs. 28 control	60. 8 ± 6.0/61.88 ± 7.9/ 60.38 ± 6.2	25.58 ± 4.8/27.20 ± 5.2/ 25.35 ± 3.4	H NMR	serum	9 different metabolites OP	6 (↑): Cholesterol, Leucine, isoleucine, Lactate, Unsaturated lipids, Allantoin 3 (↓): Tyrosine, Choline, Taurine	7
Zhang et al., 2022 [44]	China	case-control	120 OP vs. 80 control	71/70	NA/NA	LC-MS/MS	serum	(↑) NEOs and their metabolites		7
LIM et al., 1997 [32]	Korea	case-control	34 ON vs. 25 control	56.8 ± 0.4/57.2 ± 0.4	23.15 ± 0.36/24.38 ± 0.36	GC-MS	urinary	18 estrogen metabolites:		7
Qi et al., 2016 [36]	China	case-control	67 OP vs. 114 ON vs. 79 control	58.37 ± 4.78/57.03 ± 4.53/ 54.43 ± 4.9	23.52 ± 3.39/23.56 ± 3.05/ 24.75 ± 3.21	GC-MS	serum	12 different metabolites between low BMD and control 5 free fatty acids (LA, Oleic acid, AA and 11, 14-Eicosadienoic acid) correlations with BMD		8
Zhao et al., 2018 [45]	USA	case-control	65 OP vs. 71 control	31.2 ± 4.9/31.8 ± 55.3	21.9 ± 2.5/29.7 ± 8.6	LC-MS	serum	14 metabolites, 7 amino acids and amino acid derivatives, 5 lipids (including three bile acids), and 2 organic acids were significantly associated with the risk for low BMD		7
Yu et al., 2018 [37]	China	case-control	77 OP vs. 92 ON vs. 71 control	57.97 ± 4.07/56.72 ± 4.79/ 54.71 ± 4.81	23.12 ± 3.08/23.01 ± 2.98/ 24.73 ± 3.14	GC–MS	Urine	17 different metabolites		8
You et al., 2014 [46]	China	cross-sectional study	Premenopausal: 134 OP vs. 349 control Postmenopausal: 77 OP vs. 41 control	Premenopausal: 44.7 ± 0.29/44.9 ± 0.19 Postmenopausal: 52.5 ± 0.29/50.7 ± 0.47	Premenopausal: 21.2 ± 0.27/22.5 ± 0.17 Postmenopausal: 21.8 ± 0.56/24.3 ± 0.60	GC–MS	blood	7 different metabolites	2 (↑): Acetate, Glutamine 5 (↓): Lactate, Acetone, Lipids, VLDLs, Glucose	9
Mei et al., 2020 [27]	China	case-control	Discovery set: 83 OP vs. 205 ON vs. 413 control Replication set: 107 OP vs. 68 ON vs. 103 control	Discovery set: 63.0 ± 9.1/59.0 ± 10.8/ 52.9 ± 12 Replication set: 70.3 ± 9.5/66.5 ± 13.9/ 62.6 ± 12.7	Discovery set: 22.8 ± 2.9/24.2 ± 3.3/ 24.7 ± 3.2 Replication set: 22.4 ± 3.7/23.2 ± 3.2/ 24.3 ± 3.7	LC-MS	blood	47 different metabolites (13 amino acids, 2 carboxylic acids, 14 glycerophospholipids, 3 purines and purine derivatives, 7 sphingolipids, and 8 others)		9
Miyamoto et al., 2018 [47]	Japan	case-control	33 OP vs. 46 control	39–61	NA/NA	LC/MS	serum	24 different metabolites		8

* (↑): Increased expression; (↓): Decreased expression; NA/NA: Not available.

**Table 2:** Metabolites altered in ON and OP.

Category	Metabolites	Variation Trend	Reference
Amino Acids	glutamine	↓ *	Wang et al. [33]
		↑ *	Zhao et al. [45]
		↑	You et al. [46]
		↑	Miyamoto et al. [40,47]
	hydroxyproline	↑	Wang et al. [33]
		↑	Miyamoto et al. (2017) [40]
		↓	Deng et al. [41]
		↑	Miyamoto et al. (2018) [47]
	gly-gly	↓	Miyamoto et al. (2017) [40]
		↓	Kou et al. [43]
		↓	Miyamoto et al. (2018) [47]
	cystine	↓	Miyamoto et al. (2017) [40]
		↓	Zhao et al. [45]
		↓	Miyamoto et al. (2018) [47]
	taurine	↓	Pontes et al. [35]
		↑	Zhao et al. [45]
		↓	Yu et al. [37]
Lipid Metabolites	PC	↑	Aleidi et al. [34]
		↑	Cao et al. [42]
		↑	Kou et al. [43]
	LPC	↑	Wang et al. [33]
		↓	Deng et al. [41]
		↓	Cao et al. [42]
		↑	Kou et al. [43]
		↑	Miyamoto et al. (2018) [47]
	SM	↓	Cao et al. [42]
		↓	Kou et al. [43]
Carbohydrate Metabolites	glucose	↓	Kou et al. [43]
		↓	You et al. [46]
Other Metabolites	lactate	↑	Kou et al. [43]
		↑	Pontes et al. [35]
		↓	You et al. [46]
	succinic	↑	Deng et al. [41]
		↑	Zhao et al. [45]
		↑	Yu et al. [37]

* ↑: Increased expression; ↓: Decreased expression.

**Table 3:** Associated metabolic pathways involved in ON/OP.

Study	Pathways	Analysis Methods
Yin et al., 2021 [48]	Bile secretion	Enrichment analysis of KEGG signaling pathway
	Secondary bile acid biosynthesis	
	Cholesterol metabolism	
	Caffeine metabolism	
	Pyruvate metabolism	
	Primary bile acid biosynthesis	
Li et al., 2020 [50]	Valine, leucine, and isoleucine biosynthesis and degradation	Enrichment analysis and topology analysis
	Aminoacyl-tRNA biosynthesis	
	Glycolysis or Gluconeogenesis	
	Glycerophospholipid metabolism	
	Glyoxylate and dicarboxylate metabolism	
	TCA cycle	
	Taurine and hypotaurine metabolism	
Guo et al., 2022 [51]	Tryptophan metabolism	Enrichment analysis of KEGG signaling pathway
	Glutathione metabolism	
	Phospholipase D signaling pathway	
	Arginine, proline with alanine metabolism	
Aleidi et al. 2021 [34]	Histidine metabolism	The pathway analysis module
	Aminoacyl-tRNA biosynthesis	
	Glyoxylate and dicarboxylate metabolism	
	Biosynthesis of unsaturated fatty acids	
Deng et al., 2021 [41]	Lipids pathways	NA
Cao et al., 2021 [42]	Choline metabolism	The bubble diagram of pathway enrichment analysis
	Glycerophospholipid metabolism	
	Retrograde endocannabinoid signaling	
	Linoleic acid metabolism	
	Alpha-linolenic acid metabolism	
	Arachidonic acid metabolism	
Kou et al., 2022 [43]	Glucose metabolism	Database searching (KEGG) and consulting relevant literature
	Amino acids metabolism	
	Choline metabolism	
	Inflammatory response	
Zhao et al., 2018 [45]	Alanine, aspartate, and glutamate metabolism	MetaboAnalyst 3.0 software
	Butanoate metabolism	
	Taurine and hypotaurine metabolism	
	Aminoacyl-tRNA biosynthesis	
	Glutathione metabolism	
	Primary bile acid biosynthesis	
	Glycine, serine, and threonine metabolism	
Yu et al., 2018 [37]	Taurine metabolism	MetaboAnalyst 3.0 software
	β-alanine metabolism	
	Galactose metabolism	
	TCA cycle	
	Proparoate metabolism	
	Nitrogen metabolism	
	Butanoate metabolism	
Miyamoto et al., 2018 [47]	TCA cycle	NA
	Urea cycle	
	Pentose phosphate pathway	

## Data Availability

The data that support the findings of this study are available from the corresponding author upon reasonable request.

## Supplementary Materials

The following supporting information can be downloaded at: https://www.mdpi.com/article/10.3390/nu15234895/s1, Table S1: Search strategy. Table S2: The NOS assessment scale for every study. Table S3: The list of abbreviations.
