# Supplementary material for: Distinct Metabolites in Osteopenia and Osteoporosis: A Systematic Review and Meta-Analysis
Source: Nutrients. 2023 Nov 23;15(23):4895. doi: 10.3390/nu15234895 (PMC10708105; doi:10.3390/nu15234895)
Supplement: Supplementary file 1 [file nutrients-15-04895-s001.zip › nutrients-2627937-supplementary.pdf]

Supplemental Files

Table S1 Search strategy.

Table S2 The NOS assessment scale for every study.

Table S3 The list of abbreviations.

Table S1 Search strategy

Database	Search number	Query
PubMed		((((Osteopenia) OR (((((((((‘Metabolic Bone Diseases’[Title/Abstract]) OR (‘Bone Disease, Metabolic’[Title/Abstract])) OR (‘Metabolic Bone Disease’[Title/Abstract])) OR (Osteopenia[Title/Abstract])) OR (Osteopenias[Title/Abstract])) OR (‘Low Bone Density’[Title/Abstract])) OR (‘Bone Density, Low’[Title/Abstract])) OR (‘Low Bone Densities’[Title/Abstract])) OR (‘Low Bone Mineral Density’[Title/Abstract])) OR (‘Bone Diseases, Metabolic’[Title/Abstract]))) OR (Osteoporosis)) OR ((((((((((((((((((Osteoporoses[Title/Abstract]) OR (‘Osteoporosis, Post-Traumatic’[Title/Abstract])) OR (‘Osteoporosis, Post Traumatic’[Title/Abstract])) OR (‘Post-

		Traumatic Osteoporoses'[Title/Abstract])) OR ('Post-Traumatic Osteoporosis'[Title/Abstract])) OR ('Osteoporosis, Senile'[Title/Abstract])) OR ('Osteoporoses, Senile'[Title/Abstract])) OR ('Senile Osteoporoses'[Title/Abstract])) OR ('Osteoporosis, Involutional'[Title/Abstract])) OR ('Senile Osteoporosis'[Title/Abstract])) OR ('Osteoporosis, Age-Related'[Title/Abstract])) OR ('Osteoporosis, Age Related'[Title/Abstract])) OR ('Bone Loss, Age-Related'[Title/Abstract])) OR ('Age-Related Bone Loss'[Title/Abstract])) OR ('Age-Related Bone Losses'[Title/Abstract])) OR ('Bone Loss, Age Related'[Title/Abstract])) OR ('Bone Losses, Age-Related'[Title/Abstract])) OR ('Age-Related Osteoporosis'[Title/Abstract])) OR ('Age Related Osteoporosis'[Title/Abstract]))

		OR (‘Age-Related Osteoporoses’[Title/Abstract])) OR (‘Osteoporoses, Age-Related’[Title/Abstract]))) AND ((Metabolomics) OR (((((((((((((((((((((((((((((((Metabolomic) OR (Metabonomics)) OR (Metabonomic)) OR (Metabonomic)) OR (Metabolit)) OR (metabolome)) OR (h nmr)) OR (‘nuclear magnetic resonance spectroscopy’)) OR (‘proton nmr’)) OR (‘proton nuclear magnetic resonance’)) OR (‘gas chromatogra’)) OR (‘gc ms’)) OR (‘gas chromatograph-mass spectrometry’)) OR (‘gc tof ms’)) OR (‘gas chromatography/time-of-flight mass spectrometry’)) OR (‘liquid chromatogra’)) OR (‘lc ms’)) OR (‘liquid-chromatography mass spectrometry’)) OR (‘tq ms’)) OR (‘triple quadrupole mass spectrometry’)) OR (uplc))

		OR (‘ultra performance liquid chromatograph’)) OR (‘ultra-performance liquid chromatograph’)) OR (‘high performance liquid chromatograph’)) OR (‘high-performance liquid chromatograph’)) OR (‘uhplc-ms/ms’)) OR (‘ultra-high performance liquid chromatography/tandem mass spectrometry’)) OR (‘uplc qtof ms’)) OR (‘ultraperformance liquid chromatography quadruple time-of-flight mass spectrometer’)) OR (‘uhplc tq ms’)) OR (‘ultra-high performance liquid chromatography triple quadrupole mass spectrometry’)))
EMBASE	#1	'osteoporosis'/exp
	#2	'osteoporosis':ti,ab,kw OR 'osteoporoses':ti,ab,kw OR 'osteoporosis, involutional':ti,ab,kw OR 'senile osteoporosis':ti,ab,kw OR 'osteoporosis, senile':ti,ab,kw OR 'senile

		osteoporoses':ti,ab,kw OR 'osteoporoses, senile':ti,ab,kw OR 'age-related osteoporoses':ti,ab,kw OR 'bone loss, age-related':ti,ab,kw OR 'osteoporosis, age related':ti,ab,kw OR 'age-related bone losses':ti,ab,kw OR 'osteoporoses, age-related':ti,ab,kw OR 'bone loss, age related':ti,ab,kw OR 'age-related bone loss':ti,ab,kw OR 'age-related osteoporosis':ti,ab,kw OR 'age related osteoporosis':ti,ab,kw OR 'osteoporosis, age-related':ti,ab,kw OR 'bone losses, age-related':ti,ab,kw OR 'osteoporosis, post-traumatic':ti,ab,kw OR 'post-traumatic osteoporosis':ti,ab,kw OR 'osteoporosis, post traumatic':ti,ab,kw OR 'post-traumatic osteoporoses':ti,ab,kw
	#3	#1 OR #2
	#4	'osteopenia'/exp

	#5	'Bone Diseases, Metabolic':ti,ab,kw OR 'Metabolic Bone Diseases':ti,ab,kw OR 'Metabolic Bone Disease':ti,ab,kw OR 'Bone Disease, Metabolic':ti,ab,kw OR 'Low Bone Mineral Density':ti,ab,kw OR 'Bone Density, Low':ti,ab,kw OR 'Low Bone Densities':ti,ab,kw OR 'Osteopenias':ti,ab,kw OR 'Low Bone Density':ti,ab,kw OR 'Osteopenia':ti,ab,kw
	#6	#4 or #5
	#7	#3 OR #6
	#8	'metabolomics'/exp
	#9	'metabolome':ab,ti OR 'h nmr':ab,ti OR 'nuclear magnetic resonance spectroscopy':ab,ti OR 'proton nmr':ab,ti OR 'proton nuclear magnetic resonance':ab,ti OR 'gas chromatogra':ab,ti OR 'metabolomic':ab,ti OR 'metabonomics':ab,ti

		OR 'metabonomic':ab,ti<br>OR 'metabolomic*':ab,ti<br>OR 'metabonomic*':ab,ti OR 'metabolit*':ab,ti<br>OR 'gc ms':ab,ti OR 'gas chromatograph-mass spectrometry':ab,ti OR 'gc tof ms':ab,ti OR 'gas chromatography/time-of-flight mass spectrometry':ab,ti OR 'liquid chromatogra':ab,ti OR 'lc ms':ab,ti OR 'triple quadrupole mass spectrometry':ab,ti<br>OR 'uplc':ab,ti OR 'ultra performance liquid chromatograph':ab,ti OR 'ultra-performance liquid chromatograph':ab,ti OR 'high performance liquid chrormatograph':ab,ti<br>OR 'ultraperformance liquid chromatography quadruple time-of-flight mass spectrometer':ab,ti OR 'uhplc tq ms':ab,ti<br>OR 'ultra-high performance liquid chromatography triple quadrupole mass spectrometry':ab,ti

	#10	#8 or #9
	#11	#7 and #10
	#12	#7 and #10 and [embase]/lim AND [humans]/lim
The Cochrane Library	#1	MeSH descriptor: [Bone Diseases, Metabolic] explode all trees
	#2	(‘Bone Diseases, Metabolic’ or ‘Metabolic Bone Diseases’ or ‘Metabolic Bone Disease’ or ‘Bone Disease, Metabolic’ or ‘Low Bone Mineral Density’ or ‘Bone Density, Low’ or ‘Low Bone Densities’ or ‘Osteopenias’ or ‘Low Bone Density’ or ‘Osteopenia’):ti,ab,kw
	#3	#1 or #2
	#4	MeSH descriptor: [Osteoporosis] explode all trees
	#5	(‘Age-Related Bone Loss’ or ‘Age Related Osteoporosis’ or ‘Age-Related Osteoporoses’ or ‘Age-Related Osteoporosis’ or ‘Osteoporosis, Age-Related’ or ‘Bone Loss, Age-Related’ or ‘Bone Loss,

		Age Related' or 'Bone Losses, Age-Related' or 'Age-Related Bone Losses' or 'Osteoporoses, Age-Related' or 'Osteoporosis, Age Related' or 'Post-Traumatic Osteoporosis' or 'Osteoporosis, Post-Traumatic' or 'Osteoporosis, Post Traumatic' or 'Post-Traumatic Osteoporoses' or 'Osteoporoses' or 'Senile Osteoporosis' or 'Osteoporoses, Senile' or 'Osteoporosis, Involutional' or 'Senile Osteoporoses' or 'Osteoporosis, Senile'):ti,ab,kw
	#6	#4 or #5
	#7	#3 or #6
	#8	MeSH descriptor: [Metabolomics] explode all trees
	#9	('Metabonomic' or 'Metabonomics' or 'Metabolomic'):ti,ab,kw
	#10	#8 or #9
	#11	MeSH descriptor: [Metabolome] explode all trees
	#12	('Metabolomes' or 'Profiles, Metabolic' or

		'Metabolic Profile' or 'Profile, Metabolic' or 'Metabolic Profiles'):ti,ab,kw
	#13	#11 or #12
	#14	MeSH descriptor: [Gas Chromatography-Mass Spectrometry] explode all trees
	#15	('Gas Chromatography-Mass Spectrometry' or 'Gas Liquid Chromatography Mass Spectrometry' or 'Gas-Liquid Chromatography-Mass Spectrometry' or 'Chromatography-Mass Spectrometry, Gas-Liquid' or 'Chromatography, Gas Liquid Mass Spectrometry' or 'Spectrometry, Gas-Liquid Chromatography-Mass' or 'Chromatography, Gas-Liquid-Mass Spectrometry' or 'Spectrometry-Gas Chromatography, Mass or Mass Spectrometry Gas Chromatography' or 'Gas Chromatography Mass Spectrometry' or 'Chromatography, Mass Spectrometry-Gas' or 'Spectrometry, Gas Chromatography-Mass' or

		‘Chromatography, Gas Mass Spectrometry’ or ‘Spectrometry, Mass-Gas Chromatography’ or ‘Spectrometries, Mass-Gas Chromatography’ or ‘Spectrum Analysis, Mass Gas Chromatography’ or ‘Chromatography-Mass Spectrometry, Gas’ or ‘Chromatography, Gas-Mass Spectrometry’ or ‘Spectrum Analysis, Mass-Gas Chromatography’ or ‘GCMS or Spectrometry, Mass Gas Chromatography’ or ‘Mass Spectrometry-Gas Chromatography’):ti,ab,kw
	#16	#14 or #15
	#17	MeSH descriptor: [Chromatography, High Pressure Liquid] explode all trees
	#18	(‘High-Performance Liquid Chromatographies’ or ‘Chromatography, High Performance Liquid’ or ‘Chromatography, High Speed Liquid’ or ‘Liquid Chromatography, High-Performance’ or

		'HPLC' or 'Chromatography, Liquid, High Pressure' or 'High Performance Liquid Chromatography' or 'High-Performance Liquid Chromatography' or 'Chromatography, High-Performance Liquid'):ti,ab,kw
	#19	#17 or #18
	#20	MeSH descriptor: [Magnetic Resonance Spectroscopy] explode all trees
	#21	('Magnetic Resonance Spectroscopy' or 'Resonance Spectroscopy, Magnetic' or 'Spectroscopy, MR' or 'MR Spectroscopy' or 'Spectroscopy, Magnetic Resonance' or 'Magnetic Resonance Spectroscopies' or 'In Vivo NMR Spectroscopy' or 'NMR Spectroscopy, In Vivo or Spectroscopy, NMR' or 'Spectroscopies, NMR' or 'NMR Spectroscopy' or 'NMR Spectroscopies' or 'Spectroscopy, Nuclear Magnetic Resonance' or 'Resonance, Nuclear Magnetic' or

		‘Magnetic Resonance, Nuclear’ or ‘Nuclear Magnetic Resonance’ or ‘Magnetic Resonance’ or ‘Resonance, Magnetic’):ti,ab,kw
	#22	#20 or #21
	#23	#10 or #13 or #16 or #19 or #22
	#24	#7 and #23
Web of Science	#1	TS=(‘Osteopenia’ OR ‘Metabolic Bone Diseases’ OR ‘Bone Disease, Metabolic’ OR ‘Metabolic Bone Disease’ OR’ Osteopenias’ OR ‘Low Bone Density’OR ‘Bone Density, Low’ OR ‘Low Bone Densities’ OR ‘Low Bone Mineral Density’ OR ‘Bone Diseases, Metabolic’)
	#2	TS=(‘Osteoporosis’ OR Osteoporoses’ OR ‘Osteoporosis, Post-Traumatic’ OR ‘Osteoporosis, Post Traumatic’ OR ‘Post-Traumatic Osteoporoses’ OR ‘Post-Traumatic Osteoporosis’ OR ‘Osteoporosis, Senile’ OR ‘Osteoporoses, Senile’ OR ‘Senile

		Osteoporoses' OR 'Osteoporosis, Involutional' OR 'Senile Osteoporosis' OR 'Osteoporosis, Age-Related' OR 'Osteoporosis, Age Related' OR 'Bone Loss, Age-Related' OR 'Age-Related Bone Loss' OR 'Age-Related Bone Losses' OR 'Bone Loss, Age Related' OR 'Bone Losses, Age-Related' OR 'Age-Related Osteoporosis' OR 'Age Related Osteoporosis' OR 'Age-Related Osteoporoses' OR 'Osteoporoses, Age-Related')
	#3	#1 or #2
	#4	TS=('Metabolomics' OR 'Metabolomic' OR 'Metabonomics' OR 'Metabonomic' OR 'Metabonomic' OR 'Metabolit' OR 'metabolome' OR 'h nmr' OR 'nuclear magnetic resonance spectroscopy' OR 'proton nmr' OR 'proton nuclear magnetic resonance' OR 'gas chromatogra' OR 'gc ms' OR 'gas chromatograph-mass spectrometry' OR 'gc tof

		ms' OR 'gas chromatography/time-of-flight mass spectrometry' OR 'liquid chromatogra' OR 'lc ms' OR 'liquid-chromatography mass spectrometry' OR 'tq ms' OR 'triple quadrupole mass spectrometry' OR 'uplc' OR 'ultra performance liquid chromatograph' OR 'ultra-performance liquid chromatograph' OR 'high performance liquid chrormatograph' OR 'high-performance liquid chrormatograph' OR 'uhplc-ms/ms' OR 'ultra-high performance liquid chromatography/tandem mass spectrometry' OR 'uplc qtof ms' OR 'ultraperformance liquid chromatography quadruple time-of-flight mass spectrometer' OR 'uhplc tq ms' OR 'ultra-high performance liquid chromatography triple quadrupole mass spectrometry')
	#5	#3 and #4
WanFang Data		(主题:('骨量减少') or 主题:('骨质疏松症')

		or 题名或关键词:(‘绝经后骨质疏松症’) or 题名或关键词:(‘骨密度’) or 题名或关键词:(‘骨代谢’) or 题名或关键词:(‘骨痿’) or 题名或关键词:(‘骨痹’) or 题名或关键词:(‘骨枯’) or 题名或关键词:(‘骨质疏松’) or 题名或关键词:(‘原发性骨质疏松’) or 题名或关键词:(‘骨质丢失’))and (主题:(‘代谢组学’) or 题名或关键词:(‘代谢组’) or 题名或关键词:(‘核磁共振’) or 题名或关键词:(‘proton NMR’) or 题名或关键词:(‘质子NMR’) or 题名或关键词:(‘GC-MS’) or 题名或关键词:(‘气相色谱-质谱联用’) or 题名或关键词:(‘GC-TOF-MS’) or 题名或关键词:(‘气相色谱-飞行时间质谱’) or 题名或关键词:(‘LC-MS/液相色谱-质谱联用’) or 题名或关键词:(‘TQ MS’) or 题名或关键词:(‘三重四极杆液质联用’) or 题名或关键词:(‘UPLC’) or 题名或关键词:(‘超高效液相色谱’) or 题名或关键词:(‘HPLC/高效液相

		色谱’) or 题名或关键词:(‘UHPLC-MS/MS/超高效液相色谱-串联质谱’) or 题名或关键词:(‘UPLC-QTOF-MS/超高效液相色谱-四极杆-飞行时间串联质谱’) or 题名或关键词:(‘UHPLC-TQ-MS’) or 题名或关键词:(‘超高效液相色谱三重四极杆质谱’))
CNKI		(SU %= '骨质疏松症' OR TKA = '骨量减少' OR TKA = '绝经后骨质疏松症' OR TKA = '骨密度' OR TKA = '骨代谢' OR TKA = '骨痿' OR TKA = '骨痹' OR TKA = '骨枯' OR TKA = '骨质疏松' OR TKA = '原发性骨质疏松')AND (SU %= '代谢组学' OR TKA = '代谢组' OR TKA = 'NMR' OR TKA = '核磁共振' OR TKA = 'proton NMR' OR TKA = '质子 NMR' OR TKA = 'GC-MS' OR TKA = '气相色谱-质谱联用' OR TKA = 'GC-TOF-MS' OR TKA = '气相色谱-飞行时间质谱' OR TKA = 'LC-MS' OR TKA = '液相色谱-质谱联用' OR TKA = 'TQ MS' OR

		TKA = '三重四极杆液质联用' OR TKA = 'UPLC' OR TKA = '超高效液相色谱' OR TKA = 'HPLC' OR TKA = '高效液相色谱' OR TKA = 'UHPLC-MS/MS' OR TKA = '超高效液相色谱-串联质谱' OR TKA = 'UPLC-QTOF-MS' OR TKA = '超高效液相色谱-四极杆-飞行时间串联质谱' OR TKA = 'UHPLC-TQ-MS' OR TKA = '超高效液相色谱三重四极杆质谱')

Table S2 The NOS assessment scale

The NOS assessment scale for case-control.

Study		Selection				Comparability	Exposure			Scores
		S1	S2	S3	S4	C1	E1	E2	E3	
2021	Yin et al. [48]	1	1	1	0	1	1	1	1	7
2020	Zhu et al. [49]	1	1	0	1	1	1	1	1	7
2020	Li et al. [50]	1	1	0	0	1	1	1	1	6
2022	Guo et al. [51]	1	1	1	1	1	0	1	1	7
2022	Yin et al. [38]	1	1	0	0	1	1	1	1	6
2003	Poor et al. [39]	0	1	1	1	1	0	1	1	6

2019	Wang et al. [33]	1	1	0	1	2	1	1	1	8
2017	Miyamoto et al. [40]	0	1	0	0	2	1	1	1	6
2021	Aleidi et al. [34]	1	1	1	1	1	1	1	1	8
2021	Deng et al. [41]	1	1	1	1	2	1	1	1	9
2021	Cao et al. [42]	1	1	0	1	1	1	1	1	7
2022	Kou et al. [43]	1	1	0	1	2	1	1	1	8
2019	Pontes et al. [35]	1	1	1	0	1	1	1	1	7
2022	Zhang et al. [44]	1	1	1	0	1	1	1	1	7
1997	LIM et al. [32]	0	1	1	1	2	1	1	0	7
2016	Qi et al. [36]	1	0	1	1	2	1	1	1	8
2018	Zhao et al. [45]	1	0	1	0	2	1	1	1	7
2018	Yu et al. [37]	1	1	0	1	2	1	1	1	8
2020	Mei et al. [27]	1	1	1	1	2	1	1	1	9
2018	Miyamoto et al. [47]	1	1	0	1	2	1	1	1	8

Note:

S1: Is the case definition adequate?

S2: Representa-tiveness of the cases.

S3: Selection of Controls.

S4: Definition of Controls.

C1: Comparability of cases and controls on the basis of the design or analysis.

E1: Ascertainment of exposure.

E2: Same method of ascertainment for cases and controls.

E3: Non-Response rate.

The modified Newcastle-Ottawa Quality Assessment Scale for cross-sectional study.

Study		Selection			Comparability	Outcome and Analysis				Scores
		S1	S2	S3	C1	OA1	OA2	OA3	OA4	
2014	You et al. [46]	1	1	1	1	1	1	1	1	8

S1: Representativeness of the sample.

S2: Sample size.

S3: Non-response rate.

C1: Between respondents and non-respondents.

OA1: Assessment of outcome of hypersensitivity.

OA2: Reporting of point estimate (prevalence).

OA3: Reporting of the measure of variability for the point estimate.

OA4: Accounting for correlation between multilevel units.

Table S3 The list of abbreviations

Full name	Abbreviation
Osteoporosis	OP
bone mineral density	BMD
Osteopenia	ON
dual-energy X-ray absorptiometry	DXA
osteoblast	OB
osteoclastic	OC
Bone turnover markers	BTM
procollagen type I amino terminal peptide	PINP
C-terminal telopeptide of type I collagen	CTX-I
Chinese databases of China National Knowledge Internet	CNKI
body mass index	BMI
Newcastle–Ottawa Scale	NOS

Traditional Chinese Medical	TCM
glycylglycine	gly-gly
phosphatidylcholine	PC
Lysophosphatidylcholine	LPC
sphingomyelin	SM
cardiolipin	CL
phosphatidic Acid	PA
bile acid	BA
insulin-like growth factor 1	IGF-1
Triglycerides	TG
diacylglycerols	DG
oxidized low- density lipoprotein	ox-LDL
bone marrow mesenchymal stem cells	BMSCs
